# Editorial

**Published:** 1841-12-18

**Authors:** 


THE MEDICAL EXAMINER.

PHILADELPHIA, DEC. 18, 1841.

It appears that, in the report of the debate in the Philadelphia Medical Society, on the subject of vaccination, published in our 48th number, we have been misunderstood to infer that the process recommended by Dr. Benjamin H. Coates was expressly designed to draw blood from the part subjected to the operation, in order to render it more effectual. That gentleman merely recommends the removal of a visible disk of the entire thickness of the cuticle in preference to making a mere slit, as usually directed. The process recommended *by the lecturer* of that evening was not intended to be claimed as originating with himself. It was mentioned to him by Dr. J. W. Moore, and may have been very probably derived from the process of Dr. B. H. Coates. The last named practitioner also objects to the manner of our statement that he thought an imperfect vaccination might yield considerable protection against small pox. He states that his inference was based upon the almost universal employment of the scab in this country, "a resource well known to render the vaccination imperfect in the eyes of the severer Europeans;" yet as the history of variolous epidemics in this country does not show any deficiency of protection here, when compared with European results, "the inference is unavoidable, and may fairly be extended to other grades of imperfection in the process."

Many of our distant subscribers have recently inquired if any essential change is contemplated in the tone and character of the Examiner in the new series to be commenced in January next. They express satisfaction with the journal as heretofore conducted, and, in general, deprecate the proposed curtailment in its size. We find, too, the impression here and there entertained that the journal is to pass entirely into other hands—the editors whose names are now prefixed to it relinquishing all active participation in conducting it. We wish to correct these misconceptions. No radical change is intended in the character of the journal. It is the object of the editors to maintain the practical tone which has heretofore distinguished it, while they hope to give it increased efficiency and spirit in the same direction, by an accession of strength in the editorial department. Drs. Biddle and Gerhard will continue to be co-editors of the journal, in conjunction with Dr. Reynell Coates, who will assume the duties of acting editor from the commencement of the new series. The diminution in the price of subscription, will not be followed by a corresponding diminution in the amount of matter furnished. Each number of the new series will contain sixteen octavo pages, but the cover will be omitted, which now subjects subscribers to double rates of postage.

The Examiner, we may, we think, venture to say, will be found to be a compendium of whatever is most valuable in the progress of medicine, surgery, and the collateral sciences. Communications to our columns from original sources, supported by a thorough analysis of the American medical journals, will present a faithful record of the improvements which originate in our own country. The European journals are in our hands almost immediately after publication, regularly transmitted through the steamships. Selections from these, to be in most instances annotated by the editors, will occupy a large space in the journal. The most distant reader will thus, within a short period after the publication of the European periodicals, be in possession of what is best worth reading, carefully winnowed from the entire mass. We shall give regular clinical reports from the hospitals of Philadelphia, and we intend to extend them to those of other cities. Notices and reviews of new publications will be found as heretofore. The subjects of medical politics and ethics, now rapidly rising in interest, will be temperately but thoroughly discussed. To medical education, a topic much neglected, we hope to awaken the particular attention of our readers. Altogether independent of cliques and institutions—neither the creation nor organ of any school—our position is a guarantee that we shall treat these subjects with a single view to the best interests of the profession at large.

The journal has another claim upon the confidence of the profession. It is owned as well as edited by physicians. It can never be made the puffing herald of a publishing house, nor can feelings or interests without the pale of the profession, be brought to bear upon its course. Thus isolated—having the ample resources of a medical metropolis at command—we may fairly hope, with an increase of editorial strength, to publish an independent and useful journal. Aiming mainly to make it a record of valuable information, collated from all available sources, the staple matter of its columns will be purely scientific. In the discussion of collateral topics, which, though perhaps less immediately interesting to all readers, are of vital importance to the general interests of our noble profession, we shall be brief—always bearing in mind that we are writing chiefly for the working practitioner.

